# High-speed dual color fluorescence lifetime endomicroscopy for highly-multiplexed pulmonary diagnostic applications and detection of labeled bacteria

**DOI:** 10.1364/BOE.10.000181

**Published:** 2018-12-12

**Authors:** Ettore Pedretti, Michael G. Tanner, Tushar R. Choudhary, Nikola Krstajić, Alicia Megia-Fernandez, Robert K. Henderson, Mark Bradley, Robert R. Thomson, John M. Girkin, Kevin Dhaliwal, Paul A. Dalgarno

**Affiliations:** 1 Institute of Biological Chemistry, Biophysics and Bioengineering, School of Engineering and Physical Sciences, Heriot–Watt University, Edinburgh EH14 4AS, UK; 2 Scottish Universities Physics Alliance (SUPA), Institute of Photonics and Quantum Sciences, School of Engineering and Physical Sciences, Heriot–Watt University, Edinburgh EH14 4AS, UK; 3 EPSRC Proteus Hub, Centre for Inflammation Research, Queen’s Medical Research Centre, University of Edinburgh, Edinburgh EH16 4TJ, UK; 4 Institute for Integrated Micro and Nano Systems, School of Engineering, University of Edinburgh, Edinburgh EH9 3FF, UK; 5 EaStChem, School of Chemistry, University of Edinburgh, Edinburgh EH9 3FJ, UK; 6 Department of Physics, University of Durham, Durham DH1 3LE, UK; 7 Currently with the Leibniz-Institute für Astrophysik Potsdam, Potsdam, Germany; 8 Currently with the University of Dundee, School of Science and Engineering, Dundee, UK

## Abstract

We present a dual-color laser scanning endomicroscope capable of fluorescence lifetime endomicroscopy at one frame per second (FPS). The scanning system uses a coherent imaging fiber with 30,000 cores. High-speed lifetime imaging is achieved by distributing the signal over an array of 1024 parallel single-photon avalanche diode detectors (SPADs), minimizing detection dead-time maximizing the number of photons detected per excitation pulse without photon pile-up to achieve the high frame rate. This also enables dual color fluorescence imaging by temporally shifting the dual excitation lasers, with respect to each other, to separate the two spectrally distinct fluorescent decays in time. Combining the temporal encoding, to provide spectral separation, with lifetime measurements we show a one FPS, multi-channel endomicroscopy platform for clinical applications and diagnosis. We demonstrate the potential of the system by imaging SmartProbe labeled bacteria in *ex vivo* samples of human lung using lifetime to differentiate bacterial fluorescence from the strong background lung autofluorescence which was used to provide structural information.

## 1. Introduction

Fiber based optical endomicrosopy (OEM) is a minimally invasive technique used in pulmonary medicine to access the alveolar space [1–3]. Typically, OEM takes advantage of the autofluorescence from the elastin, collagen, porphyrins, flavin adenine dinucleotide (FAD) and nicotinamide adenine dinucleotide (NADH) present in the lung to provide high contrast, *in situ* cellular and sub-cellular-level images of the lung [1, 4]. Such approaches have been applied to pulmonary medicine but as yet with limited clinical adoption due to the lack of molecularly focused applications. An area of potential future progress involves coupling OEM visualization with molecularly targeted probes (SmartProbes) that could detect pathological processes.

An emerging application involves using chemical SmartProbes to sense inflammation [5], fibrosis [6] and bacterial infection [2]. These approaches involve identifying bacterial colonies by specifically targeting them with fluorescent labels so that during imaging they are distinct from the inherent background autofluorescence. Ideally fluorescent labels are spectrally separated from autofluorescence which is exploited for navigation. Instruments capable of such multi-wavelength endoscopic imaging [7] have been commercially available for some time. We have previously demonstrated a novel multi-channel fluorescence platform designed specifically for endomicroscopy for pulmonary medicine [1, 4]. Furthermore, we have also developed selective bacterial fluorescent probes that enable detection and differentiation of bacteria in the human lung [2, 8]. However, fluorescence endomicroscopy, like all fluorescence imaging modalities, is typically limited to 2 or 3 channels restricted by the need to avoid spectral overlap, therefore requiring tight bandwidth, filtering and imaging constraints. These challenges are exacerbated in the lung tissue due to the high intensity green background autofluorescence generated from intrinsic elastin and collagen.

Fluorescence lifetime imaging (FLIM) is an alternative, well established technique able to discriminate different molecules through fluorescent decay lifetime. The potential of FLIM, using the inherent fluorescence lifetime of the probes in contrast to the autofluorescence signal, is well known in the field of optical microscopy, including applications for tissue and clinical use [9–12]

Fluorescence species with distinct lifetimes, typically ranging from 1-10 ns, can be distinguished independent of their emission wavelength. When combined with multi-color spectral imaging, FLIM provides an additional selection process, increasing the number of distinct imaging channels. Furthermore, FLIM is generally insensitive to intensity variations or fluorophore density, making it a robust modality for distinguishing subtle changes. However, a major limitation is imaging speed. Single-photon counting FLIM typically requires scanning methods to accommodate complex time-tagged single-photon counting detection, and significant photon counts to construct meaningful lifetime histograms at each image pixel.

Fluorescence lifetime imaging endomicroscopy through an imaging fiber-bundle has previously been demonstrated, although limited in imaging speed or timing resolution. Kennedy et al. [13] used a 30,000 core coherent imaging fiber and were able to a measure lifetimes down to 89 ps but with an acquisition time between 10 and 100 seconds per image frame. Bec et al. demonstrated a system [14] composed of a single scanning fiber with a moving probe able to record at a 30 Hz rate but requiring 500 images to produce the results shown in the paper, equivalent to 16 seconds acquisition per frame. Yankelevich et al. [15] used a single scanning fiber, to obtain sub-nanosecond precision lifetime but with an acquisition time of 9.8 seconds per frame. Gated intensified CCD techniques offer fiber endoscopic FLIM at high frame rates, such as demonstration at 60 FPS *in vivo* [16]. However 128 frames were needed to obtain sufficient signal to noise ratio and the time resolution was limited to 200 ps.

In all cases the advantage of FLIM over conventional imaging is primarily dependent on the ability to distinguish separate fluorescence lifetimes, the sensitivity of which is dependent on photon counts, the number of channels in the decay and fitting algorithms. Time correlated single-photon counting (TCSPC) is the most accurate technique for reproducing the true decay times [17], but is inherently slow, typically delivering a single image frame in 10s of seconds.

A system that could potentially deliver higher frame rate in endomicroscopy is described by Poland et al [18]. Their system is a confocal microscope that uses a "Megaframe" camera (MF32) based on a 32×32 SPAD array developed by the University of Edinburgh and STMicroelectronics [19, 20]. The SPAD array has a very low fill factor (1%) however, a spatial light modulator (SLM) synthesizes 8×8 beamlets that are scanned across the sample, before the resulting fluorescence is descanned and directed onto an 8×8 selection of the SPAD array with near 100% fill factor. The resulting 256×256 pixel images were recorded at 10 seconds per frame. However, the speed of acquisition remains limited and the use of the spatial light modulator complicates the optical setup rendering a potential clinical instrument bulky, due to the use of a Ti–Sapphire laser, and in need of complex alignment procedures.

In this paper we present an alternative approach that uses an entire MF32 SPAD array as a single, parallel SPAD detector, to reduce detection dead time and increase count rate and thus frame rate. We demonstrate 4 channel, one frame per second fluorescence imaging through a combination of dual channel FLIM and dual color, temporally dephased photon-counting. This method of multiplexing colors in the time domain was also described elsewhere [15], the parallel detection architecture applied here enables this technique at higher rates without photon pileup [21]. We demonstrate future clinical potential of the endomicroscope by imaging *ex vivo* human lung with labelled *E.coli* bacteria compared to healthy lung.

**Fig. 1:**
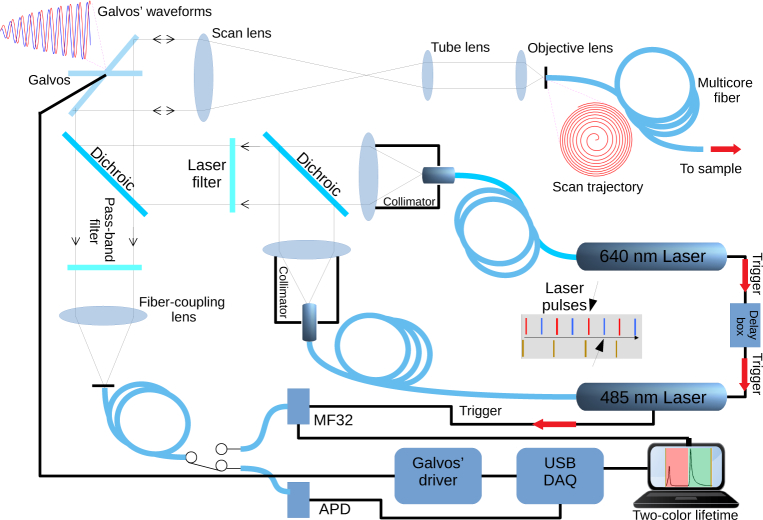
Optics diagram and block diagram of the laser-scanning endomicroscope. A USB controlled DAQ generates the scanning waveforms and acquires the analog signal from the APD when in pure intensity scanning mode. The MF32 camera for FLIM data acquisition is controlled through a separate USB interface. Center right shows the trigger signal path originating in the 640 nm laser, passing through a delay box, triggering the 485 nm laser and finally triggering the MF32 camera. The emission dichroic and excitation filters are the XF454 set from Horiba. The schematic shows a raw histogram as acquired from the MF32 camera, showing the red fluorescence decay and the temporally shifted green fluorescence decay (bottom right).

## 2. Description of the instrument

A schematic of the multiplexed endomicroscope is shown in Fig. 1. Two picosecond pulsed laser sources (480 and 640 nm, Picoquant GmbH Germany) are driven at a repetition rate of 20 MHz. The trigger output of the 640 nm laser source drives the 480 nm source through an analog and programmable delay box to introduce 25 ns delay (half the pulse period), additionally triggering the MF32 camera to start the TCSPC acquisition. The two spectrally distinct fluorescence signals from green and red excitation are thus separated in time through this temporal offsetting of the two excitation lasers. The two laser sources are fiber coupled into two collimators (Thorlabs F240FC), whose output are combined via a dichroic (FF506-DI03-25X36, Semrock USA) before passing through a multi-band laser filter (Omega XF454 set) with cut-off 10 nm above each laser wavelength to clean the emission. The resulting beam is then scanned using a pair of close coupled galvos (ThorLabs GVS202).

The resulting scanned beam is conveyed through a CLS–SL visible scan lens (400–750 nm, Thorlabs, EFL 70 mm) and an ITL200 infinity corrected tube lens (Thorlabs, f 200 mm), to an Olympus systemplan fluorite imaging objective (RMS10X–PF × 10, 0.3 NA) that focuses the excitation light onto a multi-core fiber. The fiber has ≈ 30,000 cores, with sizes varying from 5–10 microns (Fujikura, Japan). The laser scan system is achromatic for the two excitation wavelengths but the imaging fiber is not. To mitigate this the system is aligned such that coupling is equally compromised for both excitation wavelengths.

The fluorescence is collected back from the distal end of the fiber-bundle and the returning light is collected by the objective and passes back through the optical system to be descanned, before being transmitted by the multi-band dichroic filter and pass-band filter set (Omega XF454) to ensure no laser light enters the 105 μ m 0.22 NA, multimode fiber (Thorlabs M43L01) that acts also as an optical pinhole before the detectors in combination with the fiber coupling lens (Olympus Plan N, RMS10X, × 10, 0.25 NA). The fluorescence signal, the output of the multimode fiber, can be switched to either an APD detector for 10 FPS fluorescence intensity images, or defocused to fill the MF32 camera for 1 FPS FLIM imaging.

**Fig. 2:**
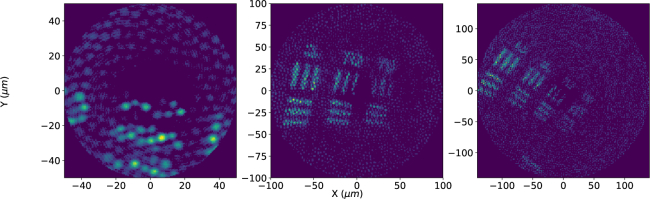
Fluorescent image sequence acquired in non time-resolved mode of a 1951 USAF resolution test chart as a standardized test of the imaging system performance at varying field of view. The sequence starts left with the highest resolution and ends right with lower resolution, zooming out of group 7, element 2. The single cores of the imaging fiber (5 μm) are visible in the highest resolution image.

The non-resonant scanning galvos are driven to achieve one frame per second imaging rate in a raster scan mode. However, by using a spiral pattern we were able to obtain frame rates in excess of 10 FPS in fluorescence imaging mode. When in the spiral-scanning mode the scan matches the circular fiber-bundle closely. An important advantage of using non-resonant galvos is that they allow us to freely control scan rates, patterns and ranges (e.g. Fig. 2). For example, the architecture described here is appropriate for performing wide field of view imaging, for focusing on specific targeted areas of tissue or for addressing single fiber cores.

To demonstrate the basic performance of the system, Fig. 2 shows a 1951 USAF resolution test chart coated on the underside with an ultra-high density of fluorescent microspheres, imaged in fluorescent mode using the APD. The sequence starts with a high resolution image of group 7, element 2 of the chart, where the single cores of the fiber are visible and zooms out to a larger field of view. Optical resolution is limited by both core spacing (around 5 μm) and by core to core coupling [22].

In FLIM modality the system uses the MegaFrame 32×32 SPAD array as a single pixel detector, rendering the detection from the 1024 individual SPAD detectors parallel. In the low photon detection limit employed here, incident photons stochastically impact on each pixel in the array and the signals from each of the 1024 SPADs detectors are then binned in a single temporal histogram of the photon arrival time for each single frame from the SPAD array producing the signal of a single pixel in the final image. Crucially this parallelization increases the maximum detectable number of photons per unit time enabling more efficient use of the fluorescently excited photons with a high counting rate. It has previously been demonstrated [23], that the use of a 8×8 SPAD array, as a single pixel detector, showed a factor 64 increase in photon rate.

For integrated CMOS SPADS, detector dead time is defined by the maximum rate at which photons can be read out from the system. In this system operation, photon counts per single detector pixel are below the maximum per detector pixel readout rate of hundreds of kHz [20] (total count rate increased to hundreds of MHz by detector parallelization over the whole array of 1024 detector pixels), while laser repetition rate is 20 MHz. If an imagined (the authors are not aware of such a device) single detector capable of 100 MHz photon count rates were used (equivalent to the maximum count rates possible here), and operated near these rates then photon pileup [21] would be overwhelming. Even at two orders of magnitude lower rates (MHz) pileup would cause perturbation of the observed photon timing statistics. This would lead to shortened observed fluorescence lifetimes, and variations of this artifact with varying numbers of photon counts. Meanwhile, at the 100 MHz maximum possible count rates in our system each detector is operating at only 0.5% of the laser repetition rate, a regime recognized to be safe from photon pileup [21].

The red and green photons reaching the detectors of the MF32 camera are separated in time due to the delay introduced in triggering the blue laser with respect to the red laser, this delay being much longer than any of the fluorescence lifetimes, as shown in Fig. 1. Counts in one color do not have an effect on the other channel (due to photon pileup) in the noted regime where counts are low per detector compared to laser repetition rate. Only in such a parallel detection scheme is high speed FLIM not in danger of photon pileup artifacts, and temporal color multiplexing becomes viable as described.

Histogramming the photon counts across all parallel pixels of our detector also combines the dark counts present. Statistical noise in a measurement is dependent on the square root of the counts. As such, parellization which simultaneously increases both photon counts and dark counts results in less noisy counting statistics than those of a count rate limited system.

For fast lifetime imaging we use a center of mass (C–O–M) algorithm [18] for real-time calculation of the exponential lifetime for both the green (485 nm excitation) and red (640 nm excitation) fluorescence. As in standard beam scanned FLIM, the photon counting occurs for a fixed dwell time for each scanned point, building up a complete image during the scan. Raw data was saved as flexible image transport system (FITS) format [24] as a sequence of 32×32 images from the MF32 with the associated scanner position enabling multiple alternative methods suitable for processing to obtain fully fitted TCSPC FLIM images. The system scan speed is limited to one frame per second by the SPAD array to FPGA transfer speed.

## 3. Results and discussion

We demonstrate the potential of the instrument by first using an example system of a multi-core fiber with 19 widely spaced cores with the distal end loaded with fluorescent microspheres, then utilizing a 30,000 core imaging fiber to view microspheres on a slide, and then viewing *ex vivo* human lung tissue to show the detection of bacteria against a strong spectrally similar fluorescence background using lifetime as the contrast mechanism. Images were obtained in 10 FPS fluorescence mode and in 1 FPSFLIM mode, in single and dual color modalities.

**Fig. 3:**
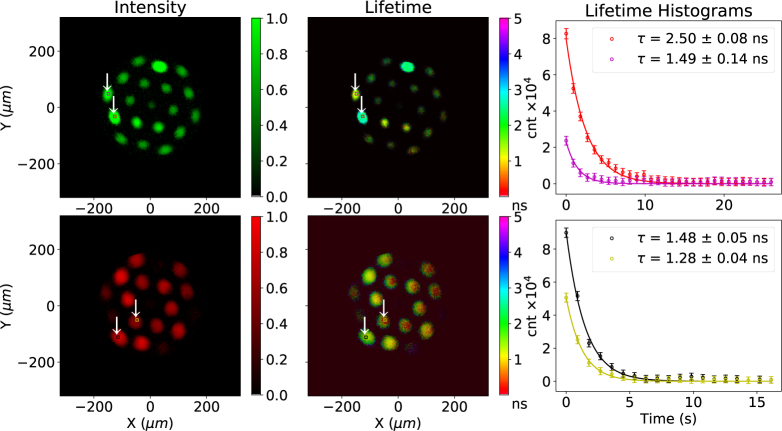
A multi-core fiber loaded with four different fluorescent microspheres acquired at 1 FPS. Top shows the green channel and bottom the red channel. The left hand column shows a fluorescence image, the center column a lifetime image. The plots show the lifetime measured from two distinct areas of 9×9 elements in the figures, marked by red and purple squares on the top row and black and yellow squares on the bottom row. The plots and the uncertainties were obtained using a single exponential fit with the scipy.optimize.leastsq module [25]. The FLIM images use the center-of-mass method [18]. The effect of varying intensity observed in the left panels is noticed as varying transparency in the middle (lifetime) panels.

### 3.1. FLIM capability test with fluorescent microspheres

To demonstrate the basic operation of the system a loaded fiber was used in place of the normal imaging fiber. Four types of microspheres were loaded on to the distal end of a 19-core multimode fiber with 10μm cores. Pits 10μm in diameter were formed by selectively etching the cores using hydrofluoric acid, into which microspheres self-locate. This configuration is used for sensing pH and other physical parameters, using procedures described in [26]. Two types of microspheres had “red” emission, two had “green” emission and each had different lifetimes. Specifically we used 10μm silica microspheres covalently bound to Fluorescein (green), TAMRA (orange), NBD (yellow) and CY5 (red) dyes. Dye loading to microspheres was performed by ourselves as described in Appendix 5.1, the number of fluorophore molecules attached is not well quantified. The observed lifetimes are likely affected by the fluorescent loading density, and can not be compared with that of free dyes. The results are shown in Fig. 3.

Figure 3 shows in the top panels the green channel and in the bottom panels the red channel. On the left side intensity images are shown while in the center lifetime images are shown. The right side of Fig. 3 shows the average histogram of the photon arrival time from the red, purple, yellow and black square areas drawn on the intensity and FLIM images.

We find that the resulting histogram obtained for each scanned point is more than adequate to produce FLIM images at the rate of one FPS. The histograms of Fig. 3 each show two exponential decays for the same wavelength of emission from the two lifetimes for the microspheres present. The green channel in the top plots shows two distinct lifetime species, 1.51 ns and 2.56 ns. The red channel also shows two distinct lifetime species, 1.47 ns and 1.29 ns. Distinction of these species is subjective in the intensity only images, but quantifiable in the lifetime images. Thus our system can easily distinguish 4 distinct species, through both spectral and temporal identification. Temporal accuracy is limited by the laser pulse width, which is approximately 100 ps [19]. This result demonstrates the ability to resolve signals from four different probes through multi-spectral FLIM at 1 FPS. In this demonstration, as the fiber cores are highly multimode, some slight variation in lifetime is observed within the individual cores due to modal dispersion for the shortest observed lifetimes. This effect is not expected to be significant, and is not observed, for the smaller cores in the imaging fiber-bundle.

The lifetime images were weighted by the fluorescent images so that low signal-to-noise (SNR) pixels are not shown. Weighting was done by modulating the alpha channel, which controls the transparency, of each FLIM image with the fluorescent image. In this way low SNR pixels become transparent and disappear from the image, but the weighting does not impinge on the color scale for the lifetimes as traditional intensity weighting would.

To demonstrate high speed FLIM we include frames from dynamic measurements in Appendix 5.2. Figure 7 shows 8 extracted frames from a 1 FPS movie, imaged under similar conditions as described for Fig. 3 but utilizing the imaging fiber. The sample consisted of mixed red and green fluorescent microspheres of the same type as described in the previous experiments. They were deposited on a microscope slide as a liquid solution and let dry. The 30,000 core imaging fiber was put nearly in contact with the slide and shifted manually using a translation stage. The microspheres in Fig. 7 are seen shifting from right to left. Each row represents a single temporal frame. Frame one is at the top. The columns from the left show a green fluorescent image, a green lifetime image, a red fluorescent image and a red lifetime image. Figure 8 shows an additional movie. We used the same sample as in Fig. 3. This time the fiber probe with fluorescent microspheres was placed in water during the capture. The red microspheres show a decrease in lifetime from 1.8 ns to about 1.2 ns after water was added. The green microspheres show a decrease of lifetime from 0.9 ns to about 0.1 ns while additional green microspheres show a decrease from 2.4 ns to 1.6 ns.

**Fig. 4:**
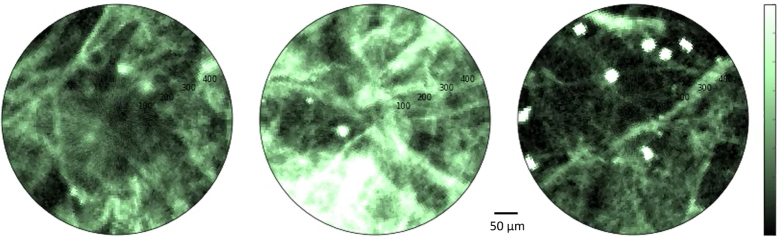
Above are three frames extracted from a 10 FPS movie from *ex vivo* lung tissue taken in in non time-resolved modality using the APD detector. Monocytes labeled with Calcein are visible in the foreground. The image intensity represents the voltage output of the APD (in response to fluorescence intensity) on the indicated color scale. The field of view is 500 μ m. Parameters were kept constant throughout the image frames giving good contrast of lung tissue, howeversome saturation of brighter features is therefore present (see Visualization 1 in the supplementary materials for the full-length movie).

**Fig. 5:**
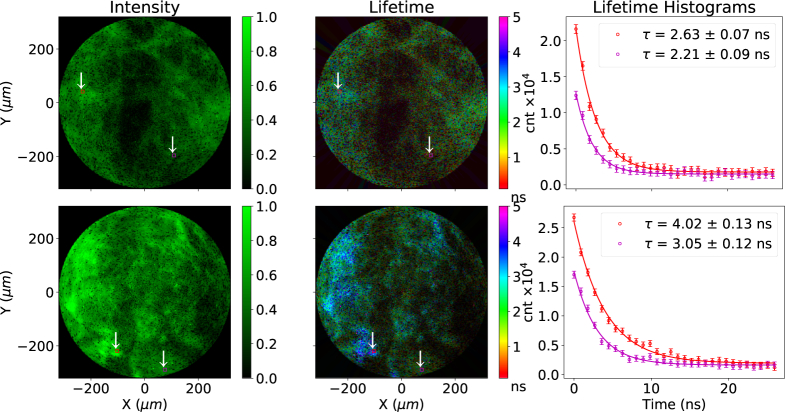
*Ex vivo* human lung tissue. The top row shows human lung and bottom row shows human lung plus *E.coli* and NBD–PMX SmartProbe. The rightmost plots show the lifetime measured from two distinct 9×9 element areas in the figures, represented by red and purple squares.

### 3.2. Ex vivo human lung tissue imaging with labeled bacteria

To demonstrate the clinical significance of the instrument, experiments were undertaken on *ex vivo* human lung tissue inoculated with labeled bacteria. Human lung samples were obtained from patients undergoing surgical resection for lung carcinoma. All lung sections used in this study were sections of normal healthy lung away from the cancerous growth. The study was approved by the Regional Ethics Committee (REC), NHS Lothian (reference 15/ES/0094), and with informed consent of the patients. Human lung tissue samples were dissected into thin, small sections (4 mm by 4 mm) and placed onto a 96–well tissue culture plate for imaging.

Figure 4 shows an extract from a 10 FPS intensity only movie of *ex vivo* lung populated with live fluorescent monocyte cells of approximately 9 μm and labeled with Calcein. (See Visualization 1 in the supplementary materials for full video). The movie was acquired using the APD single pixel detector. As previously discussed, human lung is most strongly autofluorescent in the green when illuminated with blue light mainly due to the presence of elastin and collagen in the connective tissue, therefore Calcein provides contrast by making the monocyte cells brighter. Functioning in this conventional modality the system demonstrates the observation of lung structures at 10 FPS through fiber, but it is clear that weak signals from fluorophores could be obscured by the lung autofluorescence.

**Fig. 6:**
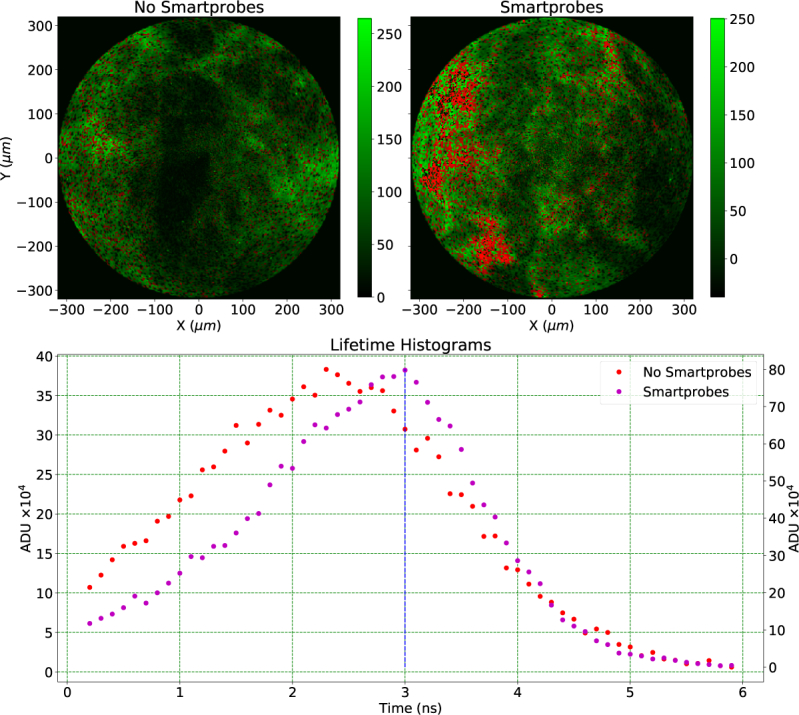
Top left is *ex vivo* lung and top right is *ex vivo* lung with NBD–PMX SmartProbe labeled bacteria, both displayed as intensity with a threshold applied based on lifetime to highlight labeled bacteria. The bottom panel shows two histograms of the lifetime distribution over the whole images. The red histogram is from the left image without SmartProbes and the purple histogram from the right image with SmartProbes. A threshold of 3 ns was applied to the FLIM data to identify regions where the SmartProbes were active. The red dots on the intensity images represent regions where lifetime was larger than 3 ns and counts were larger than 50.

### 3.3. Human lung with NBD–PMX bacterial imaging SmartProbe

Overnight cultures of *E.Coli* grown in LB broth (250 rpm, 37° C) were washed (x3) in phosphate buffered saline (PBS, Gibco) and then adjusted to an OD _595_ of 2. The bacteria were labeled with a NBD–PMX SmartProbe [2, 8] at 5μM concentration and were washed in PBS to remove any excess dye after approximately 1 minute. For imaging, pre-labelled *E. Coli* (100μL) were added to a section of lung tissue in a 96–well tissue culture plate and imaged immediately.

Figure 5 (top) shows *ex vivo* human lung tissue imaged in lifetime mode with the MF32 and C–O–M analysis described previously. Healthy lung tissue (top) and that artificially infected with *E.coli* labelled with a NBD–PMX SmartProbe (bottom) are shown. The left images show intensity only, taken from summing photon counts from the FLIM data, and the right images the intensity weighted FLIM images. All images were recorded at a rate of 1 FPS, but here 10 static frames have been combined to provide a clearer image. The rightmost plots show the average histogram of the photon arrival times from the two red and purple squares on the fluorescent and FLIM images. The intensity only images show a characteristic increase in intensity for the infected tissue, but identification of the SmartProbe labeled bacteria is challenging over the spectrally overlapping autofluorescence. In lifetime mode the distinction between bacteria (with lifetime 4.0 ns) and autofluorescence (lifetime 3.0 ns) is much clearer. Thus lifetime here acts as an excellent contrast mechanism [8].

To further exemplify this distinction, Fig. 6 shows the same intensity data from Fig. 5 with temporal thresholding applied using histograms of the lifetime present in the two FLIM images to determine the threshold. The top panel reproduces the intensity image with and without SmartProbe labeled bacteria, the bottom panel shows a binned histogram of lifetimes for each image from the FLIM data in Fig. 5. A 3 ns threshold, corresponding to the peak of lifetimes in the histogram of SmartProbe labeled data, was applied to the top images where pixels with lifetime above this threshold are then colored red and shown in both images of Fig. 6 but only notable in the right figure when SmartProbe labeled bacteria were present. With this example of thresholding, tuned to the SmartProbe lifetime, the SmartProbe labeled bacteria become immediately visible when present in the right image of Fig. 6 while being hidden in fluorescent intensity only images.

This result demonstrates the clear potential for distinguishing fluorescently labelled bacteria from the intrinsic autofluorescence of lung tissue, based on lifetime through an imaging fiber at one frame per second with simple, real time enabled processing algorithms. This method is appropriate for *in vivo* clinical use, where it is challenging to spectrally separate the SmartProbe from lung autofluorescence [2].

## 4. Conclusions

We have developed and built a dual-color, FLIM enabled laser scanning endomicroscope capable of fluorescence lifetime imaging endomicroscopy at one frame per second. The system uses a Megaframe 32×32 SPAD array detector as a single pixel detector, with all 1024 detectors contributing to the buildup of a histogram per scanned image element.

A center-of-mass algorithm with background subtraction was then used to determine the lifetime. We obtained FLIM images at a rate of one frame per second and show standard fluorescence imaging at a frame rate of 10 FPS with non-resonant galvos, using a spiral-scan pattern rather than the more conventional raster-scan. This enables a more flexible endomicroscopy system, capable of selecting regions of interest with controllable zoom, scan range and scan rate.

We have shown that the endomicroscope is capable of dual color imaging by separating the green and red channel in time using our high-speed fast recovery detector system. We have also shown that each single color channel can discriminate microspheres of different lifetime. We detected *E.coli* bacteria in *ex vivo* human lung using a fluorescent NBD–PMX bacterial imaging SmartProbe [2] that provides longer lifetime in FLIM imaging (4.02 ± 0.13 ns) from a lung backgroung of 2 to 3-ns. This demonstrates that using integrated SPAD arrays and lifetime imaging it is possible to use fluorescence lifetime as a contrast mechanism to help remove background autofluorescence in human tissue. The method is suitable for clinical use with an imaging frame rate of 1 frame per second currently limited by the data transfer speeds.

## Figures and Tables

**Fig. 7:**
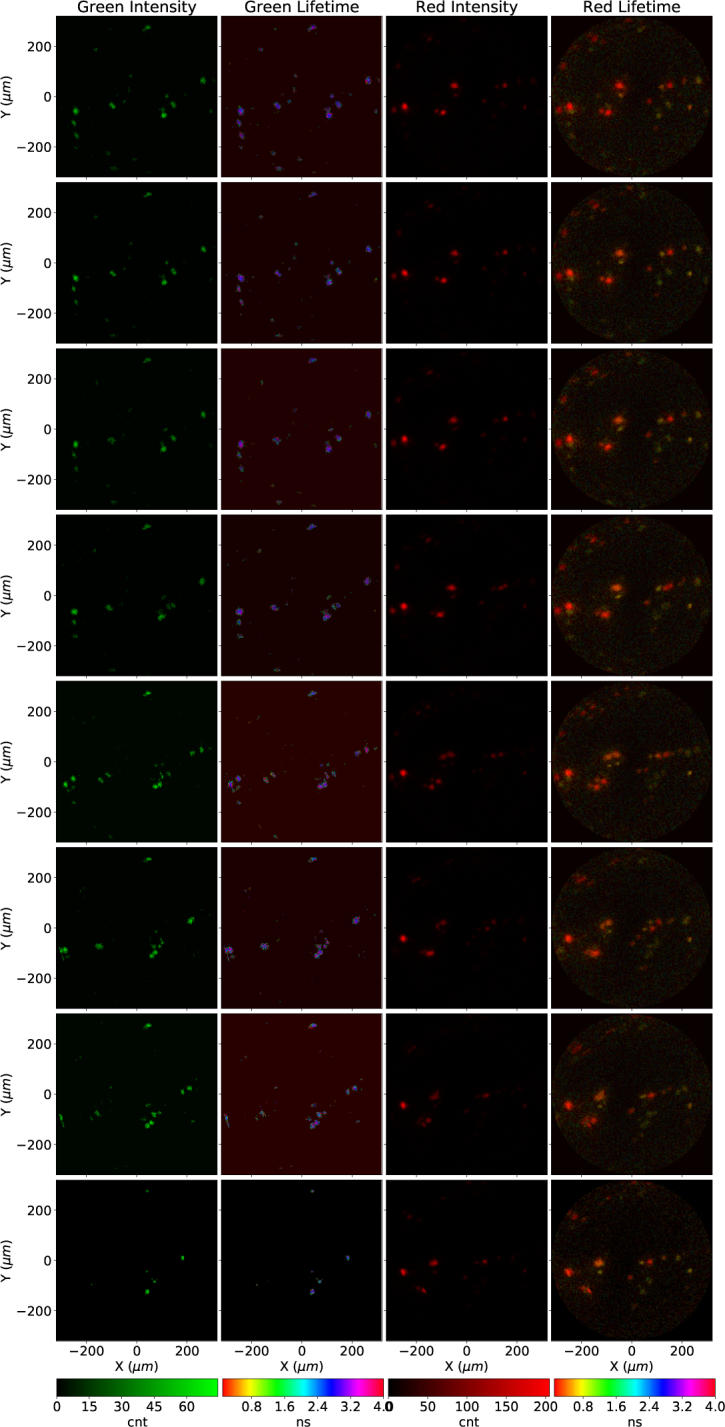
Sequence of eight images extracted from a ten-frames, 1 FPS FLIM movie. Microspheres covalently bound to Fluorescein (green), TAMRA (orange), NBD (yellow) and CY5 (red) dyes were deposited in a microscope slide. The imaging fiber was mounted on a XYZ translation stage and moved a few μm across the surface of the slide.

**Fig. 8:**
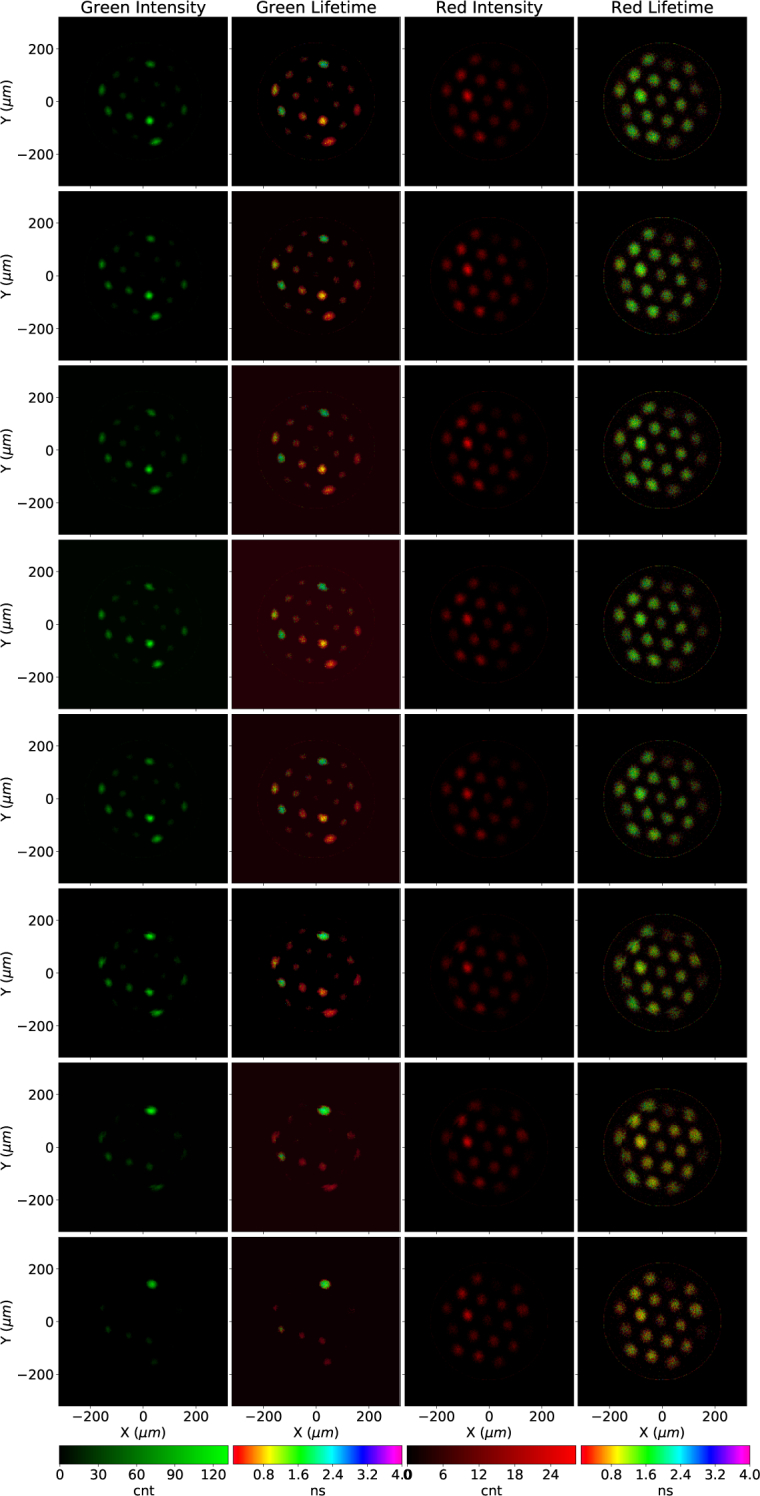
Sequence of eight images extracted from a ten-frames, 1 FPS FLIM movie. Microspheres covalently bound to Fluorescein (green), TAMRA (orange), NBD (yellow) and CY5 (red) dyes were loaded onto a multi-core fiber. The fiber was placed in water during recording, causing most microspheres to show a decrease in lifetime.

## 5. Appendix

### 5.1. Preparation of the fluorescent beads for FLIM test

To demonstrate the lifetime imaging capabilities of the endomicroscope, we used four types of fluorescent microspheres. Two with red emission, two with green emission and each with distinct lifetime. Specifically we used 10 μm fluorescent silica microspheres covalently bound to Fluorescein (green), TAMRA (orange), NBD (yellow) and CY5 (red) dyes. Amino functionalized silica particles 10 μm (Kisker Biotech Gmbh & Co) (25 mg, 0.5 mL) were centrifuged, washed with MeOH and centrifuged (×3) and washed with ether and centrifuged to finally dry at 40° C. The particles were then reacted with different fluorophores.

#### 5.1.1. Preparation of 5(6)-Carboxyfluorescein and TAMRA (5(6)-Carboxytetramethylrhodamine) silica particles

A solution containing 5(6)-Carboxyfluorescein or TAMRA (0.03 mmol) and Oxyma (0.03 mmol) in dimethylformamide (DMF, 0.42 mL) was stirred for 10 min, then N,N’–diisopropylcarbodiimide (DIC, 0.03 mmol) was added and after 1 min the solution was added to the previous particles. The reaction was kept for 2h at 50° C. The reaction mixture was centrifuged and the supernatant carefully removed. Finally, the particles were washed with DMF, 20% Piperidine/DMF, DMF, MeOH, ether and dried.

#### 5.1.2. Preparation of NBD silica particles

A solution containing 4-Chloro-7-nitrobenzofurazan (NBD-chloride) (0.03 mmol) and DIPEA (0.06 mmol) in dimethylformamide (DMF, 0.42 mL) was added to the previous particles. The reaction was kept for 6h at 50° C. The reaction mixture was centrifuged and the supernatant carefully removed. The particles were washed with DMF, MeOH, ether and dried.

#### 5.1.3. Preparation of Cy5 silica particles

A solution containing Cy5 (0.03 mmol), DIPEA (0.09 mmol) and HSPyU (0.03 mmol) in dimethylformamide (DMF, 0.42 mL) was stirred for 1h, then it was added to the previous particles. The reaction was kept for 6h at 50° C. The reaction mixture was centrifuged and the supernatant carefully removed. Finally, the particles were washed with DMF, MeOH, ether and dried.
